# Confocal microscopy: A valid approach to evaluate the three-dimensional characteristics of root-end cavities 

**DOI:** 10.4317/medoral.18440

**Published:** 2013-03-25

**Authors:** Daniel Torres-Lagares, Ramón Rodríguez-Martos, Lizett Castellanos-Cosano, Rosa Yáñez-Vico, Juan J. Segura-Egea, José L. Gutiérrez-Pérez

**Affiliations:** 1 Proffessor of Oral Surgery. Faculty of Odontology of Seville; 2 Proffessor of Restorative Dentistry. Faculty of Odontology of Seville

## Abstract

Objective: To analyze, using confocal microscope, the three-dimensional characteristics of the root-end cavity preparations completed in root apices of extracted teeth determining their area, perimeter, circularity and cavo-surface angle. Study design: Thirty-two single-rooted extracted teeth underwent endodontic treatment and apical resection. Root-end cavities were prepared according to 4 protocols, as follows: Group1, stainless steel ultrasonic tips (SST) at 33 KHz power; Group 2, SST at 30 KHz power; Group 3, diamond-coated ultrasonic tips (DCT) at 30 KHz power; and Group 4, DCT at 33 KHz power. Finally, root-end cavity was evaluated using a confocal microscope, recording its area, perimeter, circularity and cavo-surface angle. Results: The largest cavity perimeter was found in the Group 2 (4.8 ± 1.6 mm) (p & 0.05). Root-end cavities performed using SST showed larger areas than those performed with DCT (p = 0.03). The power of vibration or the tip type did not show correlation with the perimeter, circularity and cavo-surface angle of the root-end cavity (p & 0.05). Conclusions: Confocal microscopy is a useful approach to study the three-dimensional characteristics of the root-end cavity.

** Key words:**Confocal microscopy, root-end cavity, surgical root canal treatment, ultrasonic tips.

## Introduction

Periradicular surgery includes surgical debridement of pathological periradicular tissue, apical root-end resection, root-end preparation and placement of a root-end filling to seal the root canal (1). The introduction of ultrasonic retrotips in endodontic surgery, with their smaller dimensions, carried many advantages over the traditional hand pieces, including: 1) improved access to the resected root-end, 2) the tooth long axis can be followed preserving the canal morphology (1), 3) apical root-end cavities may be shaped easily, safely, and with greater precision respect to those obtained using conventional hand pieces (2), and 4) the fact that the cutting bevel on the resected root-end can be perpendicular to the canal long axis (3,4), decreasing the number of exposed dentinal tubules at the resected root surface, minimizing apical leakage (5).

The characteristics of the root-end cavity preparation have a major role in the quality of the post-surgical root seal. A hermetic seal avoids the passage of bacteria to the interface, preventing re-infection of the apex (6-8). Numerous studies have analyzed the three-dimensional characteristics and the cavo-surface angle of a dentin cavity made in dental crowns (9-12). However, in endodontic surgery these topics have not yet been analyzed and no data are available on the three-dimensional characteristics of the root-end cavity preparation performed with ultrasonic devices.

Confocal microscopy is an optical imaging technique used to increase optical resolution and contrast of a micrograph by using point illumination and a spatial pinhole to eliminate out-of-focus light in specimens that are thicker than the focal plane (13). Confocal microscopy has been used in endodontic research to investigate structural alterations in resected roots (13) and to detect bacteria in dentinal tubules (14,15). Confocal microscopy enables the reconstruction of three-dimensional structures, such as rootend cavity, from the obtained images (13).

The aim of this study was to analyze, using a confocal microscope, the three-dimensional characteristics of the root-end cavity preparations completed in root apices of extracted teeth determining their area, perimeter, circularity and cavosurface angle.

## Material and Methods

Thirty-two one-single rooted teeth without root canal treatment and restorations, with a single canal, were selected. All teeth had intact roots and closed apices and were extracted because of orthodontic and/or periodontal reasons, from subjects 18 to 50 years old. The study conformed to the ethical guidelines of the Helsinki declaration and was approved by the Ethic Committee of the University of Sevilla. After removal all periodontal tissues from the root surfaces using hand scaling, the teeth were soaked in 5% sodium hypochlorite for 30 minutes to remove periodontal remnants. Then, they were washed with distilled water and placed immediately in a solution of 5% formaldehyde for 24 hours. Radiograph was taken to assess the patency of the root canals, the absence of anatomical anomalies and to ensure the working length, set at 0.5 mm of the root apex. Teeth which root canals had irregular anatomy were excluded.

Endodontic access cavity was established by using 014 round carbide and Endo Z burs (Dentsply, New York, USA). Root canals were instrumented using a passive stepback technique, with a maximum file size of 35, using 2.5% NaOCl as irrigant. After root canal instrumentation, canals were dried with sterile paper points, and filled with gutta-percha (Guttapercha A 022E, Dentsply Maillefer, Ballaigues, Suisse) and root canal sealer (AH Plus, Dentsply, New York, USA) using a lateral condensation technique. The coronal opening for each canal was sealed with resin modified glass–ionomer cement and subsequently, all teeth were stored for 1 week at 37?C and 100% humidity to allow complete setting of the sealer.

**Figure 1:**
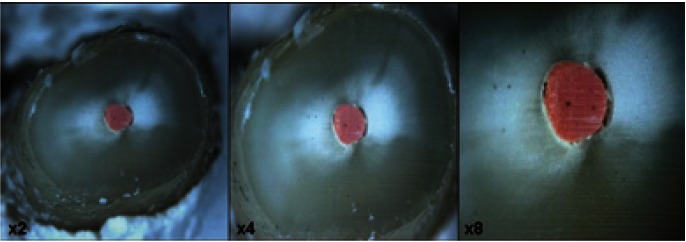
A root-end surface after resection of root apex examined at x2, x4 and x8 magnification using a stereomicroscope (Leica MZ16, zoom 16:1, Microsystems Gmbh. Wetzlar, Germany).

The root-end was resected at 3 mm of root apex using sterile water-cooled diamond blade (Diamond Saw blade, Buehler, Illinois, USA) mounted on a precision cutter (Isomet Low Speed Saw, USA) forming a 90 degree angle with the longitudinal axis of the tooth. Thereafter, each root-end surface was examined at x2, x4 and x8 magnification using a stereomicroscope (Leica MZ16, zoom 16:1, Microsystems Gmbh. Wetzlar, Germany) to ensure that no cracks were present after the root-end resection (Fig. 1).

Apical root-end cavities were prepared with the Satelec Suprasson P5 Booster (Satelec, Paris, France) under copious irrigation during 20 seconds. Four protocols were carried out, each one in eight teeth, as follows: cavities in groups 1 and 2 were prepared using stainless steel ultrasonic tips (SST), and groups 3 and 4 were prepared using diamond-coated ultrasonic tips (DCT). Two different intensities were used: maximum power (33 KHz) in groups 1 and 4, and medium power (30 KHz) in groups 2 and 3 ( Table 1). In each group, two new ultrasonic tips was employed, each one for 4 teeth.

**Table 1:**
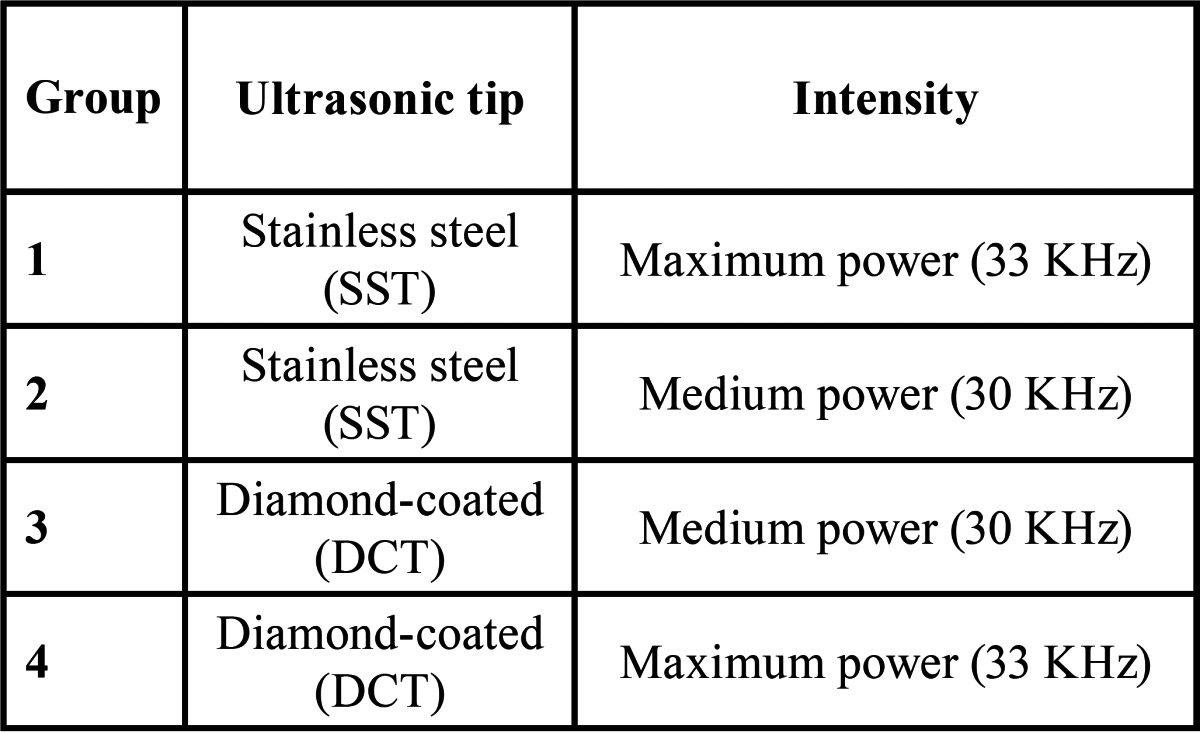
Protocols for the preparation of the root-end cavities.

Each root-end cavity was observed with a confocal microscope (Leica TCS-SP Microsystems Gmbh. Wetzlar, Germany) and the following data were recorded using the software Leica Lite (Materials package and 3D visualization): root-end cavity area, root-end cavity perimeter, root-end cavity circularity, and cavo-surface angle of the root-end cavity.

-Statistical analysis

The minimal sample size was calculated for the comparison of two independent means with nQuery Advisor® (Version 7.0). The data were tabulated in Microsoft Excel 2007 for Windows ® (Microsoft Corporation, Washington, USA) and were exported to the program SPSS ® 11 for SPSS Inc. (Chicago, USA) that performed the descriptive statistic.

Kruskal-Wallis test for multiple comparisons of continuous variables and the UMann-Whitney test were used in comparison among groups. Chi-square test was used for comparative analysis of qualitative variables. A value of p < 0.05 was considered as the significance level.

## Results

Radiographs revealed that all roots were prepared and filled to the appropriate depth. No samples were replaced or excluded from the study because of an improper root-end filling technique.  Table 2 summarizes the results of the confocal microscope evaluation of the three-dimensional characteristics of the root-end cavities.

**Table 2:**
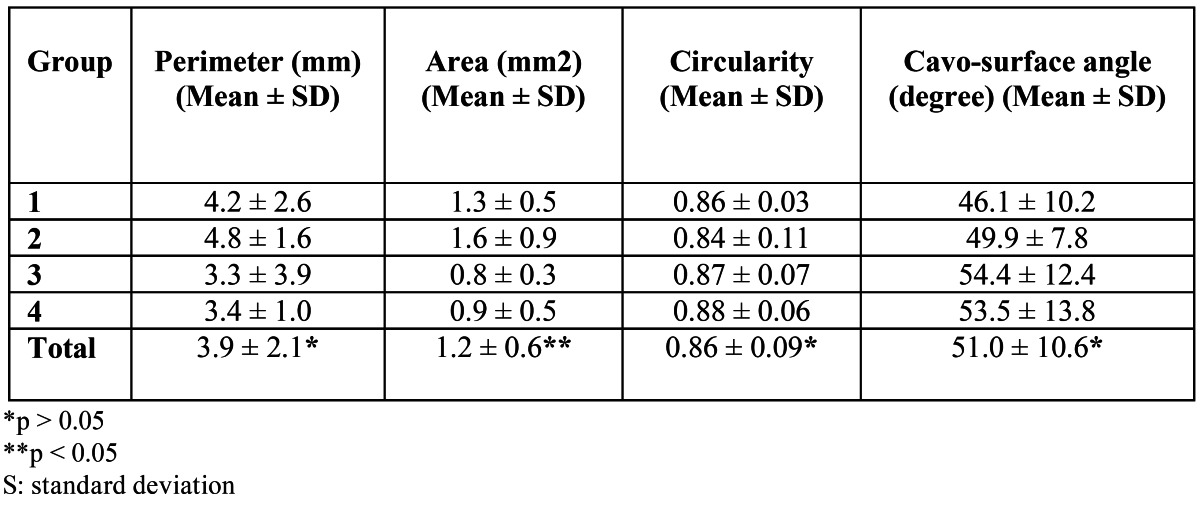
Perimeter, area, circularity, and cavo-surface angle of the root-end cavity in each experimental group after analysis with confocal microscope.

-Perimeter of the root-end cavity. Mean perimeter in the total sample was 3.9 ± 2.1 mm. The largest perimeter was found in the Group 2 (4.8 ± 1.6 mm), and the lowest one corresponded to Group 3 (3.3 ± 3.9 mm). No significant correlation between the pe-rimeter of the root-end cavity and the power of vibration or the tip type used were evident (p > 0.05).

-Area of the root-end cavity. Mean area in the total sample was 1.2 ± 0.6 mm2. The largest area was found in the Group 2 (1.6 ± 0.9 mm2), and the lowest one corresponded to Group 3 (0.8 ± 0.3 mm2). SST groups showed significantly larger areas compared to DCT groups (p = 0.03).

-Circularity of the root-end cavity. Mean circularity in the total sample was 0.86 ± 0.09, ranging 0.83 to 0.94. No correlation was found between circularity of the root-end cavity and the power or the type of ultrasonic tip used (p > 0.05).

**Figure 2:**
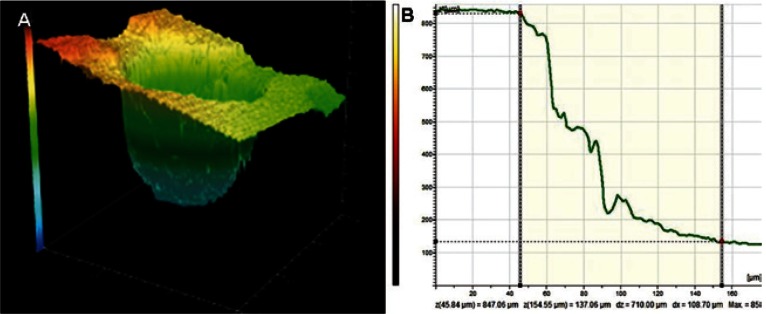
A) Three-dimensional reconstruction of a root-end cavity using confocal microscope. B) Confocal perfilometry of a root-end cavity allowing calculating the cavo-surface angle (z(x), Value of the height of the exploration in the point x; dz: interval of the height in the selected fragment; dx: value of the horizontal distance in the selected fragment; Max: maximum height in the exploration).

-Cavo-surface angle of the root-end cavity. After three-dimensional reconstruction of the root-end cavity and confocal perfilometry, cavo-surface angle was determined (Fig. 2). Mean cavo-surface angle in the total sample was 51.0 ± 10 degrees, ranging 36.5 to 66.3 degrees. The minor mean cavo-surface angle was 46.1 ± 10.2 degrees (Group 1) and the major one was 54.4 ± 12.4 degrees (Group 3). The cavo-surface angle of the cavity did not correlate with the power nor the type of the ultrasonic tip used (p > 0.05).

## Discussion

Apicoectomy combined with retrograde filling is one of the most widely performed endodontic surgical procedures. Accomplishment of apical cavity preparation with burs involves difficult access, leading to inadequate preparations. The advent of ultrasonic tips has enhanced this preparation, because of the availability of tips with different shapes and angulations (16-18).

The ideal root-end cavity preparation can be described as at least 3-5 mm deep class-I cavity, with walls parallel to the long axis of the root (18). This regularly shaped cavity should incorporate the root canal anatomy and should retain the retrograde filling material (19). Although numerous studies have analyzed the characteristics of the dentin cavities made in dental crowns, the cavo-surface angle of these cavities, as well as the adaptation of the filling material to seal the cavity (9-12), these topics have been little studied in relation to the root-end cavities performed with ultrasonic tips (20).

The present investigation has used confocal microscopy to analyze the characteristics of the root-end cavities performed using ultrasonic tips. Evaluation revealed that circularity of the root-end preparations ranged 0.83 to 0.94, the mean cavo-surface angle ranged 46.1 to 54.4 degrees, and the area of the root-end cavity was greater when SST were used.

Confocal microscopy has allowed the three-dimensional reconstruction of the rootend cavity, analyzing characteristics as perimeter, area, and circularity. In addition, confocal perfilometry has permitted to calculate the cavo-surface angle of the rootend cavity.

Confocal microscopy has been previously used in endodontics to investigate the adaptation and percentage of penetration of endodontic sealers into root dentin (21,22) and in several microbiological studies (23,24).

The results of the present study, showing the application of confocal microscopy to the study of root-end cavities, open a new field of investigation in endodontic surgery. Confocal microscopy is a useful approach to study the three-dimensional characteristics of the root-end cavity.
